# Immediate Treatment in Cases of the More Common Poisons

**Published:** 1887-08-13

**Authors:** 


### Till the Doctor Comes.

Inquiries and Communications for this column should be addressed "Physician," care of the Editor of The Hospital.]

### XXIV.—IMMEDIATE TREATMENT IN CASES OF THE MORE COMMON POISONS.

*Acids* (Nitric, Sulphuric, Hydrochloric Acids).—Give continuously plenty of alkaline fluid well diluted. Bicarbonate of soda or potash, ammonia, sal volatile, common washing soda, chalk, magnesia, or whiting, whichever may be at hand, mixed with plenty of water. Thick gruel, white of egg, and milk are also useful.

Carbolic Acid and Creosote.—Give emetics; lime water may be drunk, or a tablespoonful of sweet oil taken occasionally; stimulants will generally be necessary.

Oxalic Acid.—Give chalk, lime, or whiting. Do not give potash, soda, ammonia, or their salts.

Prussic Acid.—Death is so sudden, that usually nothing can be done. Emetics, plenty of stimulants, hot and cold douche alternately, and artificial respiration may be tried.

*Aconite.*—Give emetics, stimulants, warmth, and friction to the surface of the body.

*Alcohol* (Drunkenness).—Emetics, cold douche, keep the patient roused, flap with a cold wet towel, etc.

*Alkalies* (Potash, Soda, and Ammonia).—Give plenty of water, and in it a little vinegar, lemon juice, or orange juice; also white of egg, milk, gruel, and sweet oil.

*Antimony* (Tartar emetic).—Generally there is vomiting; if not, give an emetic. Large doses of strong coffee, milk, white of egg, may be used; stimulants, if there is much faintness.

*Arsenic.*—Emetics if necessary, plenty of magnesia, stimulants, warmth and friction to surface of body.

*Belladonna* (Deadly Nightshade).—Emetics, stimulants, coffee, hot and cold douche alternately, artificial respiration.

*Blister or Blistering Fluids.*—Emetics, barley water, white of egg, and gruel. Do not give oil.

*Chloral.*—Emetics; apply warmth and friction to the surface; keep the patient roused by speaking to him, flapping with a wet towel, etc.; artificial respiration.

*Chloroform.*—Lower the head, and commence artificial respiration; plenty of fresh air; hot and cold douche; stimulants when the patient can swallow.

*Copper Salts* (Bluestone).—Emetics if necessary; give plenty of milk and eggs, also barley water and gruel.

*Corrosive Sublimate.*—Emetics; plenty of white of egg beaten up in water, arrowroot or gruel; stimulants if necessary.

*Cyanide of Potassium*—Treatment same as for Prussic Acid. This compound, which is very deadly, and which somewhat resembles sugar, is largely used in photography, and some other processes.

*Digitalis* (Foxglove).—Emetics, plenty of stimulants, etc.; keep the patient lying down for a long time.

*Hemlock* (Conium).—Emetics, strong tea, stimulants, warmth and friction, artificial respiration.

*Laburnum Seeds*—Emetics, stimulants, hot and cold douche.

*Lead* (Sugar of Lead).—Emetics; give half an ounce of Epsom salts in water, also milk, white of egg, and barley water.

*Lunar Caustic* (Ordinary Caustic used to Warts, Corns, etc.).—Give plenty of common salt dissolved in water or milk, emetics, white of egg, barley water, etc.

*Poisonous Fungi.*—Emetics; stimulants freely; apply warmth and friction.

*Opium.*—Emetics. Keep patient walking about, pinch him, and try to rouse him in every way. Hot coffee, cold douche to head, artificial respiration.

*Phosphorus.*—Emetics; no oils or fats of any kind; white of egg, barley water, etc.

*Strychnia.*—Emetics; inhalation of chloroform if possible.

*Turpentine.*—Emetics; white of egg, barley water, milk, etc.

*White Precipitate*—Emetics; plenty of white of egg beaten up in water; arrowroot, barley water, etc.; stimulants if necessary.

Such are the poisons most likely to be met with, and their treatment. A few explanations are necessary.

Friction and artificial respiration must be applied as in the directions given under the head of drowning.

Stimulants include all wines and spirits; in bad cases preferably the latter, and often with hot water. Also sal volatile, strong smelling salts, ammonia to the nostrils, etc.

*Emetics.*—The one most usually at hand is mustard—a tablespoonful to half a pint of water; or common salt, two tablespoonfuls to half a pint of water. Sulphate of zinc, 30 grs. (half a teaspoonful) in water, is about the best of all if obtainable. It should be kept in remote country districts in the house ready weighed. Powdered ipecacuanha, same dose in water, or ipecacuanha wine, two tablespoonfuls in water, may also be taken. Tickling the back of the throat with the finger or with a feather will help to produce vomiting, and so will draughts of tepid water.

The following are poisonous compounds in common use, with the poison they contain, and in accordance with the directions for which they must be treated:—

Almond Flavour: Laurel Water: Essential Oil of Almonds: Benzol and Nitro Benzol:—(*Prussic Acid*).

Chlorodyne: Godfrey's Cordial: Syrup of Poppies: Soothing Syrup: Nepenthe:—(*Opium*).

Vermin Killers. Almost all made up of *Strychnia*.

Rat Poisons : Lucifer Matches :—(*Phosphorus*).

Emerald Green : Fly Papers :—(*Arsenic*).

Salt of Sorrel or Essential Salt of Lemons. Really an acid oxalate of potash, and must be treated like Oxalic Acid.

Spirit of Salt :—(*Hydrochloric Acid*). Sometimes kept in houses to clean brass.

